# Data on the length-weight relationship of 161 species of commercially important coral reef fishes in Tawi-Tawi islands, Southern Philippines

**DOI:** 10.1016/j.dib.2023.109537

**Published:** 2023-09-09

**Authors:** Richard N. Muallil, Ahalnida M. Tambihasan, Marylyn J. Enojario, Rosanda T. Tarabasa, Argamar A. Habibuddin, Akkil S. Injani, Mohammad Amilussin T. Ammang, Absar S. Aksa, Cleto L． Nañola Jr.

**Affiliations:** a Mindanao State University Tawi-Tawi College of Technology and Oceanography, Sanga-Sanga, Bongao, Tawi-Tawi, 7500, Philippines; b The Marine Science Institute, University of the Philippines Diliman, Quezon City, 1101, Philippines; c University of Philippines Mindanao, Mintal, Tugbok District, Davao City, Philippines

**Keywords:** Coral reef fishery, Ichthyology, Fish stock monitoring, Coral triangle, Sulu archipelago

## Abstract

This article contains the data on the Length-weight relationship (LWR) parameters play a crucial role in fisheries management, particularly in fish stock monitoring methods like fish visual census, where biomass estimation relies on the measurement of individual fish lengths in situ. Localized LWR parameters provide more accurate assessments of local fish stocks due to the influence of environmental conditions, which vary across different locations. In this study, we present the LWR parameters for 161 species belonging to 60 genera and 21 families of commercially important coral reef-associated fishes found in the waters surrounding the Tawi-Tawi islands in Southern Philippines. Among the families with at least 10 species recorded were the Serranidae or groupers (24 species), Lutjanidae or snappers (17 species), Nemipteridae or coral breams (14 species), Acanthuridae or surgeonfishes (12 species), Carangidae or jacks (12 species), Scaridae or parrotfishes (12 species), and Siganidae or rabbitfishes (10 species). Approximately 30% or 48 species exhibited a negative allometric growth pattern (b < 3), indicating that these species tend to become more slender as they increase in length. Conversely, around 14% or 23 species displayed a positive growth type (b > 3), where the fish becomes heavier as they increase in length. The majority of the species, accounting for 56% or 90 species, demonstrated an isometric growth pattern (b = 3), where the growth rate for weight and length is proportional. The LWR analysis yielded a coefficient of determination (r2) with an average value of 0.9553, indicating highly significant relationships between length and weight for all species studied (*P* < 0.001). Furthermore, this study unveiled new total length records for 27 species. Additionally, 73 species represent the first LWR records for marine fishes in the Philippines.

Specifications Table

**Table:** 

Subject	Marine Biology
Specific subject area	Fisheries science
Data format	Raw, Analyzed
Type of data	Table, Figure
Data collection	Length and weight of each fish were measured using a measuring board and digital weighing scale respectively. Analyses was performed using Microsoft Office Excel and R version 4.2.1.
Data source location	Sampling was done at the public market (tabuh) and warehouses (bodega) in Bongao, Tawi-Tawi (Figure 1). The fish samples were caught in the waters of Tawi-Tawi in the southern part of the Sulu Archipelago in southern Philippines [1].
Data accessibility	Repository name: Mendeley Data Data identification number: 10.17632/3fxyry58rb.1 (raw data) 10.17632/xch32gm2xr.1 (analyzed data) Direct URL to data: https://data.mendeley.com/datasets/3fxyry58rb/1 (raw data) https://data.mendeley.com/datasets/xch32gm2xr/1 (analyzed data) Analyzed data are also available in this article

## 1 Value of the Data

• Tawi-Tawi, part of the Sulu Archipelago and situated at the heart of the Coral Triangle, is globally recognized as a major hotspot for coral reef biodiversity, characterized by its rich and diverse marine ecosystems but increasingly threatened both by natural and anthropogenic disturbances, necessitating urgent conservation efforts.
• Localized length-weight relationship parameters are essential for accurate fish biomass estimation methods in fisheries management. Futures studies in the region can utilize these parameters for improved fish stock monitoring and fisheries management in general.
• The study presents the most comprehensive dataset on the length-weight relationship commercially important coral reef fish species in the Philippines, i.e. new total length records for 27 species and 73 first LWR records, which highlights the novelty and importance of the dataset, serving as a baseline for future research and contributing to the scientific knowledge of Philippine coral reef fisheries.

## 2 Data Description

The data presented here include both raw and analyzed data, which can be accessed at the Mendeley Data online repository. The raw data, which are presented as table in .xlsx, format, can be found at https://data.mendeley.com/datasets/3fxyry58rb/1, while the analyzed data, which are also presented as table in .docx format, is available at https://data.mendeley.com/datasets/xch32gm2xr/1. Mendeley Data is an open, free-to-use research data repository, which enables researchers to make their research data publicly available. It is a participating member of the National Institutes of Health (NIH) Office of Data Science Strategy (ODSS) GREI project.

The raw data consist of length and weight measurements of individual fishes. The length data represent the total length of individual fishes measured in centimeters from the snout to the tip of the caudal fin. The weight data represent the weight of individual fish measured in grams using a digital weighing scale.

The analyzed data comprise the LWR parameters and other information for each species. The species names were validated using Eschmeyer's Catalog of Fishes online (http://researcharchive.calacademy.org/research/ichthyology/catalog/fishcatmain.asp), and their conservation status was determined from the IUCN Red List of Threatened Species website (https://www.iucnredlist.org/). The length and weight data for each species are presented as means and ranges (minimum - maximum).

Length-weight relationship (LWR) for each species was computed following the formula, W= aL^b^ [2], where “W” is the weight (g), and “L” is the total length (cm) of the fish. “a” and “b” are the coefficient of the functional regression between L and W. The slope (b) determines the three dimensional growth of fish in length, width, and depth [3]. r^2^ represents the goodness of fit of the regression model. Some species presented in the raw data, which showed very low accuracy of regression, i.e. r^2^ values of less than 0.80, and those with less than 10 individuals that did not well represent the length range of the species that were excluded from the analyzed data [4]. The b slope for each species was statistically validated by performing student t-test against the theoretical value of 3 to confirm the observed growth pattern. Negative allometric growth pattern (b < 3) indicates that fishes grow faster in length than in weight, thereby resulting to a more slender or elongated body as they increase in length. The opposite is true for positive allometric growth pattern (b > 3) where fish becomes heavier as they grow in length. Isometric growth pattern (b =3) indicates that the growth rate for length and weight is the same. All the analyses were performed using Microsoft Office Excel and R version 4.2.1.

**Fig. 1:**
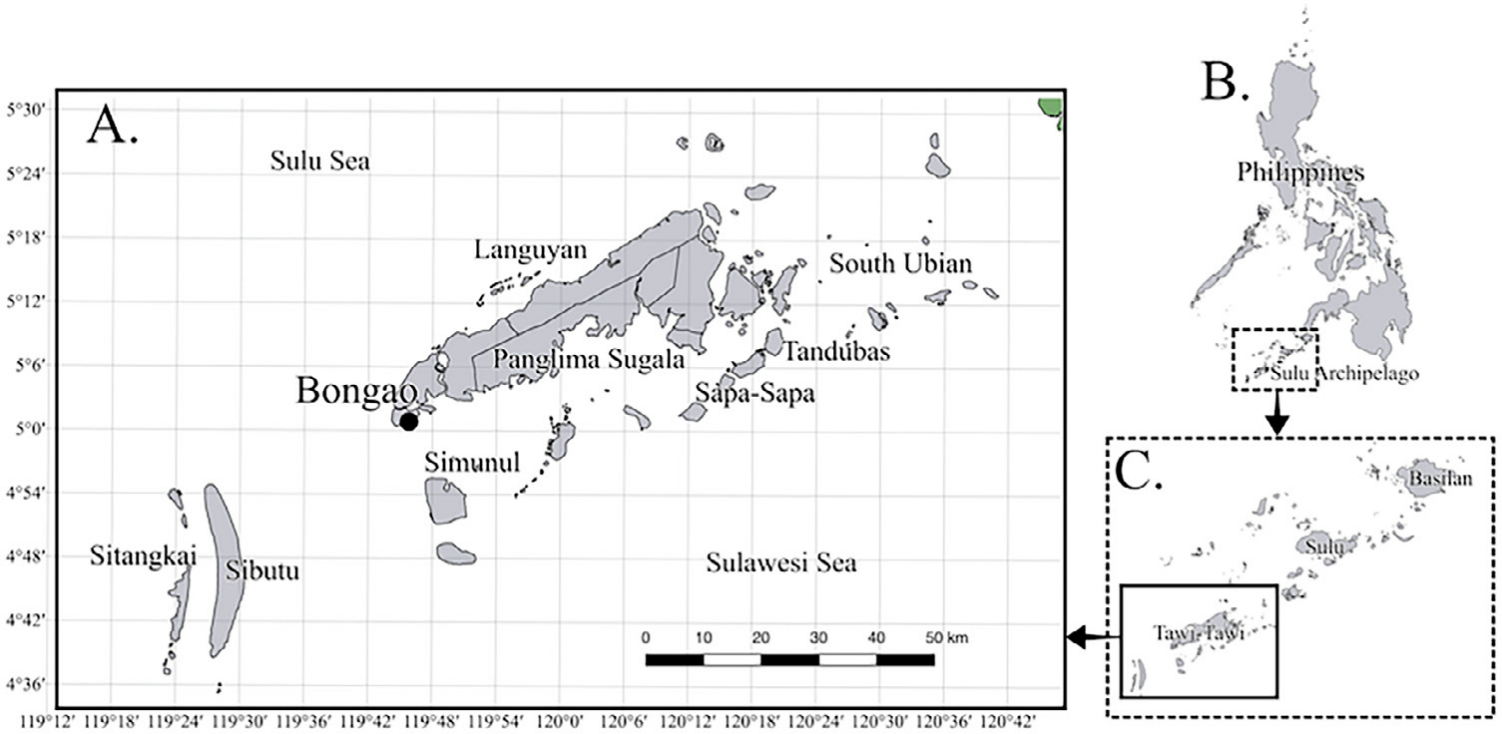
Study sites (A) Map of Tawi-Tawi province showing the location of Bongao municipality. (B) Map of the Philippines showing the location of the Sulu Archipelago, and (C) Map of the Sulu Archipelago showing the location of Basilan, Sulu and Tawi-Tawi.

Fig. 1 shows (**A**) the map of Tawi-Tawi showing Bongao and other municipalities which are the possible sources of the fish samples of the study. Also shown are portions of the Sulu Sea and Sulawesi Sea biogeographic regions. Not shown in the figure are Mapun and Turtle Islands municipalities which are further north of the Sulu Sea. It also shows (**B**) the map of the Philippines showing the location of the Sulu Archipelago, and (**C**) Map of Basilan, Sulu and Tawi-Tawi which make up the Sulu Archipelago. The map was created from QGIS version 3.22.16-Białowieża software

## 3 Experimental Design, Materials and Methods

Data collection for this study occurred between 2015 and 2022 at the public market (tabuh) and warehouses (bodega) in Bongao, Tawi-Tawi. Visits to the market were conducted opportunistically, and measurements of most fishes were made by merely borrowing them from the vendors [see 1].

Each fish was identified to the species level, and with the vendors' consent, weighed to the nearest 0.1 g using a digital weighing scale. Total length, measured from the snout to the tip of the caudal fin, was recorded to the nearest 0.1 cm. For quick measurements and to prevent spoilage of the fish, we simply photographed each fish on top of the measuring board and on weighing scale, allowing for subsequent species identification and accurate recording of length and weight measurements in the laboratory at the Mindanao State University – Tawi-Tawi College of Technology and Oceanography (MSU TCTO).

Species identification was based on morphology using the reef fish identification guides by [[5], [6], [7], [8], [9]]. For species that were difficult to identify based on morphology alone, morphometrics were employed, utilizing the FAO Species Identification Sheets for Fishery Purposes [10].

## 4 Limitations

The following are some of the limitations of the study:

• Sex identification was not possible as the fishes were only borrowed from the vendors for rapid measurements.
• The study focused on coral reef-associated fishes, which are commonly monitored in situ through non-destructive fish visual census (FVC).
• Some species had few individuals recorded at the market, i.e. less than 10 individuals, which were eventually excluded from the study.

## Ethics Statement

All fish samples included in the study were from the public market and fish warehouses in Bongao, Tawi-Tawi. The researchers did not catch/collect any fish-sample directly from the wild. Permission from vendors at the public market and operators/owners of fish warehouses were sought prior to measurements. Some samples were bought from the public market and fish warehouses.

## CRediT authorship contribution statement

**Richard N. Muallil:** Conceptualization, Supervision, Methodology, Formal analysis, Writing – original draft, Project administration, Funding acquisition. **Ahalnida M. Tambihasan:** Conceptualization, Writing – original draft, Formal analysis, Investigation. **Marylyn J. Enojario:** Investigation, Writing – review & editing. **Rosanda T. Tarabasa:** Investigation, Writing – review & editing. **Argamar A. Habibuddin:** Investigation, Writing – review & editing. **Akkil S. Injani:** Investigation, Writing – review & editing. **Mohammad Amilussin T. Ammang:** Investigation, Writing – review & editing. **Absar S. Aksa:** Investigation, Writing – review & editing. **Cleto L． Nañola Jr.:** Conceptualization, Supervision, Writing – review & editing, Project administration, Funding acquisition.

## Data Availability

Raw data of length-weight relationship parameters of commercially important coral reef fishes from the Sulu Archipelago (Original data) (Mendeley Data). Length-weight parameters of coral reef fishes from the Sulu Archipelago (Original data) (Mendeley Data).
